# Is digital health care more equitable? The framing of health inequalities within England's digital health policy 2010–2017

**DOI:** 10.1111/1467-9566.12980

**Published:** 2019-10-10

**Authors:** Emma Rich, Andy Miah, Sarah Lewis

**Affiliations:** ^1^ Department for Health University of Bath Bath UK; ^2^ School of Environment and Life Sciences University of Salford Salford UK

**Keywords:** E‐health, Health Policy, Healthism, Internet, Social determinants of health, Youth

## Abstract

Informed by a discourse analysis, this article examines the framing of equity within the UK's digital health policies between 2010 and 2017, focusing on England's development of NHS Digital and its situation within the UK Government's wider digital strategy. Analysis of significant policy documents reveals three interrelated discourses that are engaged within England's digital health policies: equity as a neoliberal imaginary of digital efficiency and empowerment; digital health as a pathway towards democratising health care through data‐sharing, co‐creation and collaboration; and finally, digital health as a route towards extending citizen autonomy through their access to data systems. It advances knowledge of the relationship between digital health policy and health inequalities. Revealing that while inclusion remains a priority area for policymakers, equity is being constituted in ways that reflect broader discourses of neoliberalism, empowerment and the turn to the market for technological solutionism, which may potentially exacerbate health inequalities.

## Introduction

Health inequalities and the social determinants of illness and disease have received growing attention within public health research, which has focused attention on policymaking as a route towards making meaningful changes to the fair distribution of healthcare services. This is crucial, as it has been found widely that social inequalities have deleterious consequences for population health (Marmot and Wilkinson 2001, Peacock *et al*. 2014, Scambler 2012). Indeed, even in countries where the reduction of health inequalities has been a stated policy priority (Smith and Kandlik Eltanani 2015), disparities persist. Numerous authors note that this is partly due to the ‘lifestyle drift’ (Popay *et al*. 2010) in which policies begin by recognising the need for upstream action to address wider social and economic determinants of health, only to be reduced to a focus on individual behaviour (Baum and Fisher 2014, Williams and Fullagar 2018).

In this context, digital health technologies have been positioned by governments around the world as central to the delivery of a fair healthcare system and ‘promise to transform healthcare systems including strategies of personal risk management, modes of treatment and practices of care’ (Petersen 2019: 22). Yet, the presumption that conversion to digital health services from an analogue world will deliver on such ambitions needs careful analysis and verification, not least there is great variation in the way in which digital health is experienced by citizens/patients. Thus, digital health encompasses web‐based solutions, mobile phone and tablet applications, the integration of artificially intelligent platforms, the utilisation of wearable devices that track biometric information and the proliferation of social media environments, each of which may have varying impacts on health care equity.

Our starting point is to argue for the need to clarify the impact of digital health on fostering health equality across different settings. To do so, we argue that there is a need to examine the policy discourse that surrounds the drive towards digital health care and focus, here, on the recent work of the UK Government, notably through its ambitions for care provision within England.^1^ Since the inception of the United Nations E‐Government survey, the United Kingdom has appeared in the Top 10 countries and has been in the Top 5 in its 2 most recent iterations (2016 and 2018), making it a country valuable to analyse. The UK has also been a primary international influence in matters of diversity and inclusion, as articulated in its various Acts of Parliament, including recently the Health and Social Care Act (2012) and the Care Act (2014). The UK has also sought to position itself as a global leader in digital health, evidenced through the implementation of various programmes since the early 2000s (Cabinet Office 2013, Department for International Trade, 2018; Department of Health and Social Care. 2014) and early digital adoption in the 1980s (National Advisory Group on Health Information Technology in England, 2016). Finally, in 2014, the UK Government also published its ‘Digital Inclusion Strategy’ (Cabinet Office, 2014a) and a Digital Inclusion Charter (Cabinet Office 2014b), which reiterate the ambition to improve equality within health and care provision, made apparent in the strategy of its Department of Health and Social Care's Executive Agency, Public Health England (Public Health England (2017). Yet, despite these initiatives, there remain key inequalities within public provision.

As such, this article considers how discourses of equity are framed in public policy on digital health within England, so as to ascertain where there are gaps and need for further development or investigations. Informed by Rizvi and Lingard (2011: 6), we examine how these policies are linked to ‘a broader set of conditions in which its meaning and significance are articulated’. In so doing, we critically analyse a decisive period in the development of the UK Government's digital health trajectory, encompassing a period of two government cycles (2010–2018), during which time a remarkable amount of investment in and discussion about digital health has taken place. Notably, since 2013, a new English body named ‘NHS Digital’ has emerged, as the ‘trading name of the Health and Social Care Information Centre (HSCIC), which was established in April 2013 by the Health and Social Care Act 2012’. In large part, telling the story of England's digital health strategy is well articulated by the story of NHS Digital, the work of which is now the focal point for all other plans (Health and Social Care Information Centre, 2015).

By analysing influential digital health policy documents specifically in England over this period, this article develops our understanding of how health inequalities emerge and persist, which we assert as a crucial complement to other methods of assessing inequalities, such as patient/citizen experience surveys. Indeed, presently, there is no adequate data to assess the impact of many digital health services, but the principles by which such services are designed through policy can reveal insights into how such concerns are understood. Thus, we foreground the contribution of policy analyses to our understanding of these digital health inequalities and present the findings of an analysis of UK and English governmental and policy documents as discourse.

Throughout our analysis, we highlight concerns about how digital access and equality are constituted through and absent within these discourses, questioning how this reifies or challenges the above dominant ‘policy paradigm’ (Scott‐Samuel and Smith 2015: 418) within neoliberalism which ‘restricts the ability of policy actors to image alternative, more equitable scenarios’. In this regard, while digital health is often described as a solution to various health crises, including those of widening health inequalities, such policies are being introduced within a policy climate that is persistently focused on tackling health inequalities via downstream solutions (Baum and Fisher 2014, Popay *et al*. 2010, Smith and Kandlik Eltanani 2015, Williams and Fullagar 2018). As such, our analysis examines how digital health is being positioned by and constituting these broader discourses of health inequalities. Furthermore, we examine how these policy texts establish conditions of actions and types of selfhood and subjectivities, specifically in terms of how citizens are positioned as objects of policy interventions. This article describes these current policy directions in resolving health inequalities more broadly before making the case for critical analyses of the positioning of digital health within these public health responses. Following this, we present three key discourses that emerge from our analyses, concluding with suggestions for future policy research and theorisations of digital health inequalities.

## Current limitations in public health responses to health inequalities

In recent times, ample evidence from non‐governmental and research organisations has revealed the marked and persistent health disparities among and across different social groups, which specifies the extent to which social inequalities have deleterious consequences for population health (Marmot and Bell 2012, Marmot and Wilkinson 2001, Peacock *et al*. 2014, Scambler 2012; WHO, 2008). These circumstances have led to increasing pressure on governments to respond and develop public health policies to address these gaps in provision. For example, in the UK, there was a period of focused policies intended to reduce health inequalities between 1997 and 2010, which led the UK to be ‘recognised as a global leader in health inequalities research and policy’ (Garthwaite *et al*. 2016: 459). Despite such efforts, inequalities persist and, in some cases, have widened (Bambra 2012; Mackenbach 2011). Indeed, Mackenbach (2011) notes that, although England was the first European country to pursue a systematic policy to reduce socioeconomic inequalities in health, it has failed to reach its own target of a 10% reduction in inequalities in life expectancy and infant mortality.

Although government strategies and systematic policy responses will vary, attempts to addressing population health and accompanying disparities are usually ‘characterised by a chasm between two central views of how population health may be improved through action to prevent ill health and promote health’ (Baum and Fisher 2014: 214). On the one hand, as certain chronic diseases or conditions (such as obesity) have ostensibly increased, governments have targeted individual behaviours such as physical activity, diet and smoking to address the risks associated with these conditions. However, targeting individual lifestyle and behaviour within new public health approaches (Petersen and Lupton 1996) has been heavily critiqued, not least because of its narrow focus on individual empowerment and on nudging people to change their behaviours. Conversely, perspectives which focus on broader social, cultural and economic factors, which influence and determine health outcomes, highlight the need for health policy and interventions that direct collective action.

As Baum and Fisher (2014) argue, despite the increasing evidence about social determinants of health, many governments continue to draw from behavioural explanations in developing policy responses. This common (re)framing of structural forces as matters of individual will is a tendency that is anticipated under conditions of neoliberalisation and ‘healthism’ (Crawford 1980) and is now well documented within the sociological literature as a lifestyle drift in health policy which involves a ‘tendency for policy to start off recognising the need for action on upstream social determinants of health inequalities only to drift downstream to focus largely on individual lifestyle factors’ (Popay *et al*. 2010: 148). Baum and Fisher (2014: 216) consider this lifestyle drift as ‘a by‐product of the appeal of behavioural health promotion’. Elsewhere, Williams and Fullagar (2018: 2) examine this drift through an exploration of the complexities of advanced liberal governance that help explain ‘discrepancies between policies that address health inequalities and the interventions designed to reduce them’.

## The rise and promise of digital health solutions

Alongside the increasing interest in health inequalities, there has been a significant growth in digital health technologies and their integration into health care systems. It has been argued that discourses of ‘promise’ play a crucial role in the development of such digital health policies (Petersen 2019) whereby digitality is positioned as a necessary component of all healthcare solutions. Digital health technologies are increasingly viewed by health organisations, governments and health professionals as crucial components in the advancement of preventative medicine/health care and are rationalised ‘against the backdrop of contemporary public health challenges that include increasing costs, worsening outcomes, “diabesity” epidemics, and anticipated physician shortages’ (Swan 2012: 93). Indeed, there has been a great deal of excitement among healthcare providers and governments about the potential of these technologies to develop a more effective healthcare system (European Commission, 2014) and to foster the ‘digitally engaged patient’ (Lupton 2013). As such, digital health has emerged as a priority focus in a range of UK and European Government and health organisation policies and reports (Department of health (2012a), European Commission, 2014, UK Government Digital Strategy (2013)) and consultations (European Commission public consultation, 2014) and the digital agenda is seen as a flagship initiative for public health as part of the Europe 2020 growth strategy. These policies are also assembled through a range of different bodies and affects, such as the desires of different lobby groups or citizens (e.g. quantified self movement) policymakers, health professionals and other agents.

Within the area of inequalities more broadly, not just health, Robinson *et al*. (2015: 569–570) argue that ‘digital inequality deserves a place alongside more traditional forms of inequality in the twenty‐first century pantheon of inequality’ claiming that ‘it has the potential to shape life chances in multiple ways’. Indeed, this connection between digital inequalities and other inequalities is becoming increasingly important given the trend towards digital health interventions and this is made evident in the UK Government's wider strategies on digital inclusion (Cabinet Office, 2014a,b).

As the responsibility for the prevention and management of health shift increasingly onto patients (as consumers) and to technological systems, this raises questions about the potential for digital health to widen or narrow health inequalities. Those who experience high levels of social disadvantage are at risk of experiencing the worse health outcomes, yet may also lack the access, digital skills and knowledge to make sense of digital health systems.

Petersen (2019) argues that digital health is ‘a field underpinned by promise and optimism, but accompanied by relatively little critical assessment of its social, economic, political and personal implications’. In this context, we caution against an uncritical widespread adoption digital health solutions and the assumption that digital solutions will always be better (see Rich and Miah 2014). Indeed, there is emerging evidence that some digital health technologies might worsen inequalities. For example, the use of mobile health apps and other mobile technologies to improve women's access to health resources has not shown clearly positive effects (Jennings and Gagliardi 2013). Alternatively, digital solutions might not address the needs of health services in rural areas or even save travel costs. Thus, we must ask critical questions about how digital technologies are negotiated, taken up and managed in the contexts of these broader social inequalities. It is also crucial to consider people's shifting investments in health or anxieties about technologies and surveillance, as many people may find digital solutions to be outside of their abilities to manage. Given the continued investment in digital health, a rapid response is imperative in addressing this knowledge deficit to inform the long‐term development of digital health policy and practice.

Much of the discourse around digital health and inequalities has been framed by the established notion of a digital divide, which evidences a sizeable majority who do not have access to the internet. This digital divide ‘represents inequalities across income, education and age groups, and between the most and least healthy’ (McAuley 2014: 1119). Recent research suggests there may be a lack of access to the Internet amongst populations of those with long‐term illness, health problems or disabilities (Dutton *et al*. 2013).

Such concerns are increasingly important, given that it is ‘now well understood that digital inequality and exclusion cannot be analysed apart from the offline circumstances of individuals and groups and that specific forms of digital exclusion map onto particular kinds of offline disadvantage’ (Robinson *et al*. 2015: 570). Similar approaches need to examine the social inequalities that preclude particular forms of digital health engagement, before they are exacerbated by an unscrutinised drive towards further digital health solutionism. This is especially important as digital health expands into even more complex territories, such as artificial intelligence, for which there is already a burgeoning enthusiasm within the healthcare sector. For example, the UK Health Tsar Sir John Bell advocates investment into artificial intelligence, as a crucial criterion of all future health care, saying how it could ‘save the NHS’ (Bell cited in Ghosh 2018).

## Discourse analysis of digital health policies

This article arises from a wider study on digital health and young people during which an analysis of digital health policy documents was undertaken.^2^ Our approach draws on a poststructuralist analysis of policy which foregrounds the concept of discourse. Foucault (1977: 49) describes discourses as ‘practices that systematically form the objects of which they speech … Discourses are not about objects; they do not identify objects, they constitute them and in the practice of doing so conceal their own invention. We utilise the concept of discourse to explore the ways in which equity is constructed within recent digital health policies in the UK. This draws attention to the power and privileging or constraining affects of ‘*policy as discourse’* (Ball 2015). As Maguire and Ball (1994: 6) claim; ‘Discourses thus provides for or privileges certain relationships and types of interaction, certain organisational forms and practices, certain forms of self‐perception and self‐presentation, and at the same moment, excludes others’.

Through this analysis, we address questions such as how is equity articulated and what are the implications of the ways in which this is framed? In other words, through this analysis we aim to identify the main policy positions and the discourses specifically in relation to representations of equity. Foucault (1978) identifies policy as a technology of governmentality, which constitutes and regulates conduct. In considering digital health *policy as discourse* we need consider they ways in which ‘subjects and subject positions are formed and re‐formed by policy’ (Ball 2015: 2) Through the concept of discourse, we ask which values, norms and subjectivities are being constituted through language. From this perspective, digital health policy discourse therefore provide us with ways of thinking about digital health, but also about ourselves and others; constituting subject positions through which we might come to understand ourselves as productive, healthy or informed citizens. This requires our analysis goes beyond analysing the content of a text. Instead, we draw attention to how particular articulations of equity and digital health are made possible. As Ball (2015: 6) argues ‘discourse is the conditions under which certain statements are considered to be the truth’. Our analysis thus explores the discourses which come to constrain, enable, frame and make possible ways of speaking about equity and digital health within the selected policy texts. The analysis we present below therefore focuses on how expressions of equity are articulated and justified through particular discourses. To do so, we undertook a Discourse Analysis of a selection of policy documents as detailed below.

### Document selection

The articulation of national strategic ambitions within any sector context is inherently complex, as many different organisations work towards adoption and delivery of policy. As such, to assist in the identification of relevant policy directions, documents were selected using keyword searches of policy archives on the following government websites: gov.uk and england.nhs. The search terms used on these websites included; digital health, mhealth, telehealth, health, digital literacy and inequalities. A list of search results by term was created and documents that appeared across these lists were read in more detail. If they were deemed relevant to the broad theme of digital health, then they were placed on a shortlist. Documents on this shortlist were then read, compared and discussed among the authors. In this article, we report on an analysis of the following UK documents, each of which were developed during the critical period of the UK Government's digital health trajectory described above (2010–present):

1. Department of Health (2012a) Digital Strategy: Leading the culture change in health and care (DoH, 2012a)
2. Department of Health (2012b) ‘The Power of Information: Putting Us All in Control of the Health and Care Information We Need’ (DoH, 2012b)
3. National Information Board (2014, Nov) Personalised Health and Care 2020 Using Data and Technology to Transform Outcomes for Patients and Citizens A Framework for Action
4. NHS England Publications (2016) ‘Healthy Children: Transforming Child Health information’ (NHS England, 2016).

The analysis of these documents differs from more traditional policy analysis, as it moves beyond single case policy analysis to provide cohesive comparative policy analysis, which has long been called for in the wider policy analysis literature (Taylor 1997, Walt *et al*. 2008). Our analytical approach to examining policy as discourse involved a series of steps similar to those undertaken by Carabine (2001) in her genealogical analysis of how lone motherhood was spoken of in Britain in the early 1990s.

Initially, reports were read in detail for their framing of equity and how this was assembled through broader values and discourses. To aid this process, we undertook a keyword analysis using WordSmith software. These two forms of familiarisation occurred simultaneously and invariably influenced one another. Close reading provided an opportunity to engage with the documents, ask questions of the content, gain an understanding of the social and political context in which they exist, and adjust to the differences in language useful between documents. WordSmith was used as an overviewing tool, which allowed authors to query language use in more detail and get a sense of language in context over such a vast amount of data. Keyword analysis made the qualitative analysis more approachable as a body of data. Some of the keywords analysed were; empower/empowerment, engage/engagement, adolescence/adolescents, access, management, self, individual, potential, responsibility, digital. The analysis then built on this familiarisation, concepts that were flagged as interesting were substantiated through further linguistic inquiry, comparison between documents and critical questioning of what was happening in these texts (and the concepts under analysis) in terms of social practices, actors and structures. Not all concepts could be substantiated and it became apparent that much was *missing* from these discourses. Essential in this analysis was the interplay of discourses thus, from a Foucauldian perspective, understanding how they constrained and enabled what could be said; for example how they conformed to neoliberalism, shaping the ways in which inequality can be perceived, discussed and acted on, in these documents.

The inter‐relationships between key discourses were then explored (e.g. equity and neoliberal discourses of empowerment). One of the aims of this study was to understand how subject positions were being (re)formed through these digital health policies; what expectations were being constituted in terms of the roles for citizens or ‘users’ of these technologies. Subsequently, our reading involved identifying how equity was performed through discursive strategies and how the digital *user* as subject was being imagined in relation to particular values, norms and subjectivities. This analytical process involved comparison of documents, discussion between authors and critical reflection on the analysis as it was being conducted. Our analysis focuses on how these policies impact upon equality, asking how they matter, who they exclude/marginalise and what policies can achieve. Finally, the transcripts were analysed in terms of notable absences or silences (e.g. what was not spoken) in relation to the above literature on inequalities which informed the research. This involved multiple readings but also looking across the policies which reflected a crucial period in the UK government's digital health trajectory. Thus, in addition to what was presented through reading and keyword analysis of the policy texts, we also explore the similarities, silences and anomalies. Following Carabine (2001: 281), this approach can be considered an overlapping and iterative process, taking us back and forth between data, analysis, theory and literature.

## Findings

Given the emphasis on downstream policy interventions to tackle inequality described above, we were interested in how individuals were described and positioned in the policy document sample and how these approaches were justified. Our analysis reveals three interrelated discourses drawn upon in the policy orientations towards equity; equity as a neoliberal imaginary of digital efficiency and empowerment; digital health as a pathway towards democratising health care through data‐sharing, co‐creation and collaboration; and finally, digital health as a route towards extending citizen autonomy through their access to data systems. We organise our discussion of the findings around these three discourses.

### Equity as a neoliberal imaginary of digital efficiency and empowerment

As recognised elsewhere, ‘a range of governmental processes are involved in defining a policy problem, in diagnosing deficiencies and in making promises of improvement’ (Rizvi and Lingard 2011: 8). In the context of digital health policy, given the concerns about austerity and welfare cuts, the UK government and various health organisations foreground the benefits of digital strategies for implementing low‐cost policy options to address inequalities.

Indeed, across the documents, policy investments in digital health care are justified on the basis of their ability to deliver greater efficiency of overburdened healthcare systems. Throughout, efficiency is rationalised on the basis of developing systems which also enhance empowerment through a ‘digitally engaged patient’ (Lupton 2013). The rising appeal of digital health solutions to influence individual behaviours is rationalised ‘against the backdrop of contemporary public health challenges that include increasing costs, worsening outcomes, “diabesity” epidemics, and anticipated physician shortages’ (Swan 2012: 93)

The accelerating pace of technological change offers unprecedented opportunities to interact with health and care services in ways that are convenient, cost‐effective and reliable. In taking advantage of this transformation – as many of us have already done in so many other areas of our lives – we should be confident that personal support is available when needed. (DoH, 2012b: 19)

As described above, despite the development of focused policy strategies to address public health challenges, inequalities persist within health care. Scott‐Samuel and Smith (2015: 419–420) suggest that ‘one obvious explanation for this phenomenon of ineffective political action on health inequalities is that politicians are attracted by non‐controversial relatively low‐cost policy options which can be implemented in a short timeframe’. Reflecting this logic, the policy documents are steeped in the language of cost‐effectiveness and its benefits in terms of a more efficient healthcare system. This vision for public health is set out in the DoH (2012a) digital strategy:

There are many advantages to going digital, both for users and for taxpayers. The most obvious improvement will be making public services easier to use, giving people access to services online, reducing the number of forms they need to fill in, giving people the information they need to help them in their everyday lives. (Dr Dan Poulter, Parliamentary Under‐Secretary of State for health)

Thus, one way in which equity is (re)framed is through its connection with a range of other values and neoliberal governmental techniques. Echoing a form of market liberalism, these techniques are strongly associated with the turn towards the operations of the market and its assumed efficiencies, where goods and services are seen as critical in preventing illness, managing risk as way of enhancing *health outcomes for all*:

Better use of data and technology has the power to improve health, transforming the quality and reducing the cost of health and care services […] Digital technologies are changing the way we do things, improving the accountability of services, reducing their cost, giving us new means of transacting and participating. This is more than an information revolution: it puts people first, giving us more control and more transparency. (NIB, 2014: 3)

Policy recommendations put forward are assumed to enhance opportunities by equipping patients as ‘informed consumers’ to make better choices to manage and gain control over their own health. This emphasis on prevention is clearly linked to the broader vision of the NHS, set out in its five year forward view plan (2014: 7) positioning ‘prevention’ as crucial to future health and wellbeing as an issue of ‘prevention’:

*The health and wellbeing gap*: If the nation fails to get serious about prevention then recent progress in healthy life expectancies will stall, health inequalities will widen, and our ability to fund beneficial new treatments will be crowded out by the need to spend billions of pounds on wholly avoidable illness.

To ensure sustainability, health and care needs to move from a model of late disease management to early health. Information technology plays an essential and rapidly expanding role in empowering people to take charge of their own health, by providing information, support and control. (NIB, 2014: 9)

These rationalities drive an intensified focus on self‐government, preventative medicine and the increased importance of individual responsibility to access health information. Transformation, quality of care and increased inclusion are all implied with the policy commitments of these texts. Rizvi and Lingard (2011: 10) argue that ‘the relationship between the values of equity and efficiency is not a simple one, linked to an instrumental logic’. This instrumental discourse features in the DoH Digital Strategy (DoH, 2012a):

In any sector, advances in technology help people to do things quicker, more efficiently and with better results. And launching a health information revolution that puts patients in control of their health and care information, and makes services convenient, accessible and efficient, is now a major priority for the Department of Health.

Similar to the lifestyle drift (Popay *et al*. 2010) described earlier in this article, within existing economic and political systems of neoliberalism, these digital solutions are being positioned as a tool to enhance downstream interventions focused on individual responsibility. These include the promotion of public‐facing digital health services which are deemed to both increase efficiency but also empower people, conveying expectations of the responsibilities of citizens as consumers of particular digital goods and services:

In addition, with the growing popularity and use of smartphones and tablets, the health and care system of the future will direct us, as patients and the public, towards accredited health apps to help us keep ourselves healthy and, as appropriate, manage our conditions. (DoH, 2012b: 64)

Digital health care is therefore framed through a ‘logic of choice’ (Mol 2008) whereby the concept of the patient as a customer or citizen emerges within an instrumental logic oriented towards the market and its health services and products. Many of these digital interventions transfer responsibility away from the state and onto the individual reflecting a broader neoliberal logic of empowerment as part of a focus on predictive, personalised, preventive health care.

While there may be many benefits to the development of these digital systems, they also invoke a series of critical questions about their ideological framing. Through these discourse, digital health users or patients are constituted as rational ‘consumers’ having agential capacity through which their individual behaviour is amenable to change through engagement with goods, service and information. Arguably, the articulation of these powerful discourses of empowerment, obscure broader social and economic factors, which inhibit individual's opportunity to act upon this knowledge and undertake health practices (Cohn 2014). Furthermore, it suggests that a particular agential capacity, in line with a behavioural model, where digital tools are used for the delivery of health interventions to manage behavioural change such as smoking cessation, alcohol reduction, increasing physical activity or ‘managing’ mental ill health (e.g. developing resilience).

Many of the policy documents therefore reflect the increased focus on self‐management, predicated on the assumed capacity for digital technologies to help engage people in changing or adopting new health behaviours

Our ambition is for a health and care system that enables people to make healthier choices, to be more resilient, to deal more effectively with illness and disability when it arises, and to have happier, longer lives in old age; a health and care system where technology can help tackle inequalities and improve access to services for the vulnerable. (National Information Board, 2014: 4)

Such a framing is utilised in policy discourse to judge the ‘success’ of health outcomes, whereby individual needs can be met through more tailored, precise and personalised digital One of the suggestions put forward by the Department of Health (2012b) is to ‘consider what progress the health and care system has already made and what can be learnt from other industries and the wider economy. We then set out a series of proposals that will: “enable me to make the right health and care choices” – citizens to have full access to their care records and access to an expanding set of NHS‐ accredited health and care apps and digital information services’. Tracking and monitoring may provide tailored benefits to specific communities or provide the means further support, enhancing health equity:

Information can bring enormous benefits. It is the lifeblood of good health and wellbeing, and is pivotal to good quality care. It allows us to understand how to improve our own and our family's health, to know what our care and treatment choices are and to assess for ourselves the quality of services and support available. (Department of Health, 2012b: 4).

However, it is presumed that people will make better choices, if given information about their behaviour, thus largely overlooking the structural action or upstream interventions required to provide equity of opportunity: As noted earlier, it is well recognised that a focus on changing behaviours remains a dominant and often appealing approach to developing health policies (Kelly and Barker 2016), most notably with regards to state‐funded research on the illness‐producing behaviours of people in lower socioeconomic groups (Scambler, 2012). However, Blue *et al*. (2016), Ioannou (2005) and Thompson and Kumar (2011) discuss the extent to which they constitute and are constituted by neoliberal notions of the self. Despite this, discourses of empowerment and rationalities of neoliberalism persist across these UK documents.

Although the documents analysed here vary in their terminology, this discourses present within them both promulgate neoliberal imperatives which can reducing issues of equality to a primary focus on empowering agential capacities of individuals. Particular subject positions oriented around healthy, responsible and informed citizens are thus (re)formed through these policies. These documents write about how citizens and individuals are restrained and responsible for their own health and their families. They are positioned as susceptible to prevention messages and willing to self‐manage and track their health. Moreover, patients/carers, citizens and individuals are written into these documents as compliant actors, willing to access (outside of a clinical setting) their own health data and seeking to be accessible to healthcare professionals.

### Democratising health? Data sharing, co‐creation and collaboration

A second core rationale for digital health rests on the techno‐utopian vision that digital health will have transformative effects in creating a ‘more democratic future’ (Petersen 2019: 17) where citizens have more autonomy over their health and care. Information is central to how agencies collaborate, whereby sharing digital information is considered crucial to better managing health problems and democratising health. One of the guiding principles invoked through these policies is that access to more health knowledge and data may facilitate collaboration and interaction between patients and health. This is a discourse expressed strongly in *The Power of Information* strategy:

‘The ability to share information following assessment between all the agencies involved in a child's care would greatly improve joining up of services around the child, and help parents and children better manage the child's condition and retain as much independence as possible’. (DoH, 2012b: 34)

Chapter 2 covers the information held within our individual care records. It sets out a vision in which being able to access and share our own records can help us take part in decisions about our own care in a genuine partnership with professionals. (DoH, 2012b: 7)

The integration of care through information sharing is central to the perceived value of digitisation within health care. Interoperability and openness are positioned as important features to ensure delivery across services and systems, to both enable patient access, but also enable collaboration and sharing of information.

‘This is about ensuring that information reduces, not increases, inequalities and benefits all’. (Department of Health, 2012b: 5)

One of the stated goals in this vision of digital health is to dismantle barriers between patients and healthcare professionals and moves beyond techno‐determinism through the call for a cultural shift within health care in the UK. An example given in *Department of Health* (2012b) illustrates how a digital tool can be implemented for mental ill health self‐management;

South London and Maudsley (SLaM) foundation trust has launched an online health record that gives service users meaningful access to their records as well as allowing them to contribute to the system directly. The open patient record has been developed as a web portal using Microsoft's HealthVault platform. The aim is to allow clinicians and patients to work collaboratively on care and treatment rather than it being an isolated experience. (Department of Health 2012b: 24)

However, it does not set out a blueprint for a devolution of power from healthcare professional to patient. Instead, it acknowledges the social and cultural contexts in which patient/doctor interactions take place, calling for a shift in how these interactions are conducted. Nevertheless, this document has sound principles underpinning its recommendations, attempting to advance digital health in the UK through access to information.

Reflecting a focus on ‘citizenship’, there also is recognition of the potential use of digital technologies to create more democratic health care policies. One such is example is the use of digital technology to involve the public in the policy process. In 2014, following the launch of the Government's Digital Strategy (2013), the UK Government pioneered the use of digital media, notably Twitter, to encourage pubic commentary on a draft bill. In a news story published by the government, it notes how this ‘was the first time a government department had made a draft Bill available for comment online in this way and at such an early stage in the process. The department is ‘closing the circle’ by explaining how people's comments are influencing changes to the Bill.’ Similarly, the National Information Board (2014: 4) identified one of its aims to move towards ‘more detailed work on implementation, it will prioritise co‐production with citizens, and partnership with initiatives like NHS Citizen’.^3^

The digitisation of health care has also resulted in an expanding range of agencies who are able to collate and share data. The shift towards the co‐production of health between health services and the public, is foregrounded in the NHS England (2016) *Healthy Children: Transforming Health Information*, which sets out a vision for restructuring health information services and systems for children, young people, parents and families. This outlines changes towards ‘transformed child health information services’ made up of ‘various information services exchanging data in a standardised format via a central hub. Information will flow to where it is needed, improving the experience of care and health outcomes for children, young people and their families and supporting the professionals providing that care’ (NHS England, 2016: 6). The vision for this Digital Child Health Hub, therefore brings together existing system with care information provided by others (e.g. parents) through the use of online personal health records. Autonomy is promoted as one the key benefits to this development, ostensibly achieved through young people having an online record of their own health and care issues and families having opportunities to co‐produce health.

However, as indicated argued elsewhere (Rich and Miah, 2017), the enhanced capacity for data collection and sharing raises some critical questions about the capacity for governmentality of particular social groups. As such, inequalities might arise through the utilisation of data for decision making about health care or future funding plans, raising critical questions about patient autonomy. As Rich and Miah (2017) suggest, it is necessary to examine how data might be used as ‘expert knowledge’ which far from addressing disparities, might create new inequalities through discourses of risk. Such considerations are relevant to the expansion of organisations who might be involved in the collection of health related data:

There will be specific informatics requirements to support the new public health system. These include helping local authorities to collect data that was previously collected by the NHS, for example child height and weight surveillance data to track child obesity and data to monitor delivery of the NHS Health Check programme. There is also an opportunity to improve the effectiveness and efficiency of national screening programmes by enhancing the informatics systems that drive them. Finally, the health of our children is of paramount importance to the future health of our nation. An expansion in the Health Visitor service and a series of other public health policies rely on the Child Health Information System to be effective. This system needs to be developed further to provide the best possible support for national and local child health priorities such as vaccination, commissioning care for disabled children and child safeguarding. (DoH, 2012b: 54)

It is now well established that the production of knowledge about and on people's bodies through quantified norms, can be considered to be part of a ‘biopolitics’ of populations (Foucault 1990) through which particular subjects are normalised and moralised. Yet, there are key questions about social inequalities which arise in relation to how such data/information is utilised in the development of particular health promotion programmes interventions or funding plans.

### Autonomy and access to data systems

In this final section, we examine how discourses which emphasise autonomy are assembled alongside those of democracy and empowerment in these policy texts. In part, this perhaps reflects a broader concern about health disparities that ‘people with the least amount of autonomy ‐ the least amount of control over their work conditions or other major life circumstances ‐ have the poorest health’ (Buchanan 2008:17). Digital technologies are justified in terms of being able to enhance autonomy, and thereby address this disparity, partly through providing opportunities for patients to access digital systems and take control of their own health care.

In the UK, efforts have been made to advance systems which enable users to access their health information and patient records, with that aim that ‘all patient and care records will be digital, real‐time and interoperable by 2020’ (National Information Board, 2014: 29).

Across the policy texts we analysed, there is clear evidence of the focus on developing digital literacy to enhance accessibility to health care and improve patient autonomy. One of the guiding principles of this approach is the assumption that this enhanced autonomy would therefore facilitate collaboration and interaction between patients and health care professionals;

The forward view assigns a central place to personal health records as a means of enfranchising parents, families and young people as equal partners in their care and providing a means of collaborative care. (NHS England, 2016: 19)

The primary use of information is to support high quality care. The most important source of information is the information held in our own health and care records. The information in our records can help make sure our health and care services join up efficiently and effectively, with us at their centre. Being able to access, add to and share our health and care records electronically can help us take part in decisions about our own care. (DoH, 2012b: 16)

As such, there is evidence of recognition within these documents for the need to enhance some aspects of what Sykes *et al*. (2013: 150) describe as ‘critical health literacy’

a distinct set of characteristics of advanced personal skills, health knowledge, information skills, effective interaction between service providers and users, informed decision making and empowerment including political action as key features of critical health literacy. The potential consequences of critical health literacy identified are in improving health outcomes, creating more effective use of health services and reducing inequalities in health thus demonstrating the relevance of this concept to public health and health promotion

The focus on literacy is further elaborated in the Power of Information strategy, in which it is suggested that initiatives may be needed to support individuals in developing appropriate literacy:

A partnership bringing together representatives from the voluntary sector, health and care professions and industry will consider how to make the most effective use of its combined skills, experience and resources to engage directly with us as patients and the public, increase our health literacy and support information producers to communicate effectively in ways that are meaningful to us. (DoH, 2012b: 66)

The above policy statements offer important steps towards addressing some aspects of digital inequality, particularly given the evidence that digital skills and levels of prior digital engagement (Hargittai and Shaw 2014) may preclude some people from digital health practices. Given that the digital footprint gap is widening, particularly among children (Robinson *et al*. 2015), it is likely that people's health opportunities will develop differently if such disparities are not addressed in future design of digital health. According to McAuley (2014), those who are most in need are the least likely to access and benefit from digital health interventions. Furthermore, Volandes and Paasche‐Orlow (2007) argue that poor health literacy ought to be understood as an injustice of the healthcare system given it is a risk factor for poor health outcomes. Failure to address these complexities of digital health literacy might therefore further exclude those considered most vulnerable according to identified social gradient in health outcomes associated with levels of socioeconomic conditions.

Opportunities to utilise technologies to address a range of factors which contribute to health inequalities could be more fully harnessed in future digital health policy. By this, we are referring to the broader range of material, social and cultural inequalities (Krieger *et al*. 2010, Wilkinson and Pickett 2009) and, more specifically, the opportunities to address those inequalities which are the product of relationalities of power (Bambra *et al*. 2005). For example, political action is defined as a feature of critical health literacy, yet the possibilities for digital innovation to enable such collective response is not fully explicated in these policy documents. However, literacy is reduced to a matter of developing the correct competences in order to manage individual health appropriately within a model of behaviour change. The dominant discourse slips back into a focus on the individual; for example on how digital health literacy could be used to develop functional skills to access and interpret information to support healthy lifestyle choices:

Good information and advice are only useful to us if we have some understanding of the health or care issues and options open to us, i.e. our health literacy. We know that health literacy levels are not high for many people, so initiatives such as health trainers can provide that additional advice and support required to make healthy living choices and decisions about our own care. (DoH, 2012b: 66)

Boyd (2014) and Livingstone and Helsper (2007) highlight the different ways in which particular populations, such as young people, access, use and engage with the Internet. These approaches highlight more complex understandings of digital engagement and exclusion, situated within relationalities of power and agency. Read this way, we need more nuanced approaches that attend to the experiences of users across, what Livingstone and Helsper (2007) describe as, ‘a continuum of digital inclusion’ (p 684). Thus, further critical exploration must identify how engagement with digital health technologies is shaped by sociocultural context (geographical, familial, spatial, religious, socioeconomic, cultural) and background (age, gender, digital experience). Elsewhere, NHS England (2016: 73) recommend the need to include young people in the design and development of relevant digital services:

Use the capability of a new digital platform for children's health information to deliver apps and information which are co‐created with children and young people and with Education services and which are suitable for teaching and use in schools as part of an ongoing curriculum of self‐care.

Differing stakeholders must take into account how people's prior experiences should collectively shape policy. Moreover, this difference may be exacerbated by the use of digital health technologies by key agents or carers in people's lives (carers, teachers, parents, health professionals). In this sense, rather than always offering solutions to inequalities, digital technology can distribute health through a range of relational, multiple and intersecting factors.

While there is recognition that those with greatest health needs might also be those with least opportunity to engage with digital services, there is a need for more nuanced policy recommendations which address differences in conditions within which health practices and choices are made possible (Mol 2008) There is evidence of some initial explorations of this, notably in the UK's Healthy Children Transforming Child Health (2016: 30–2) document, where young people were asked about their concerns when it comes to accessing data. The issues reported highlighted the complexity and challenges of developing digital literacy, including: questions of security, data access, data sharing, language use and data control. Given these concerns, it raises critical questions about expectations on citizens to engage with personal health records and other online information.

As such, increased literacy may also mediate the benefits of using these technologies, which then makes literacy a condition of entry. Consequently, prioritising an egalitarian form of empowerment and access seen in these documents may be detrimental to patients and their (digital) health literacy, as individuals who do not feel ready for empowerment can be overwhelmed by the responsibility this process requires. This is pertinent, as Coulter *et al*. (2014) question whether it is ethical to ask patients to discuss their lived experience, if there is no possibility of intervention. So understood, the presumed empowerment through information sharing may diminish perceived autonomy about one's health and increase feelings of resignation about the fact that knowledge cannot be acted upon. Newly empowered patients, or even healthy citizens, may have information readily available, but might be unable to proceed appropriately, as they still lack crucial medical knowledge and authority or the opportunity for particular health practices (Cohn 2014). The UK documents analysed do not explicitly caution about the misgivings of empowerment. Rather, the tone is positive and optimistic about patient empowerment and, especially, access to information.

## Conclusion

All policy language around health care presently reinforces the centrality of digital solutions but there is only sporadic attention given by authorities to matters of variation in the impact or benefit of digital health technologies for different social groups, including those who are marginalised and underprivileged. Yet, understanding this variance – or not assuming that digital solutions diminish health inequalities – has yet to be fully acknowledged as a key area of concern for policymakers (McAuley 2014). Such investigations are important especially as evidence suggests that equality is improved *only* in circumstances where participants have high levels of digital literacy (Robinson *et al*. 2015). Where this does not exist, then, the drive towards digital health solutions may exacerbate social inequalities due to the displacement of any other solution by digital solutions.

This article has presented findings from a discourse analysis of a selection of UK and English governmental and policy documents on digital health, highlighting concerns about how digital access and equality are constituted through and absent within these discourses. We identify the need to understand them in terms of how they establish conditions of actions and types of selfhood. The analysis has revealed how equity is (re)assembled through discourses of efficiency and empowerment, democracy and autonomy. As such, citizens are positioned as objects of policy interventions in ways that assume particular agential capacities, but which obscure myriad forms of social, political, cultural and economic inequalities which impact engagement with digital health.

We recognise that many of the policies we have analysed are relatively new and it will be necessary for subsequent research to track the policy effects across time and space and the conditions of possibility that are created through policy‐in‐action (Fullagar *et al*. 2015). As expressed elsewhere, ‘public policy formations that appear stable, potentially even complete, are never so settled. A great deal of hard political work is done in drawing heterogeneous elements together, forging connections and sustaining them in the face of tensions’ (Rizvi and Lingard 2011: 8). Similarly, Bansel (2015: 5) argues that ‘the multiple and often contradictory discourses, narratives, practices and experiences through which the subject of policy is governance, are embodied in ways that exceed the rationalities and ambitions of policy’ This means attending to the way in which policy knowledge travels in and around different social sites, such as families, health agencies, schools and is taken up, (re)contextualised, negotiated and resisted.

Extending this line of analysis further, there is a need for policymakers to engage with the social, cultural, geographical, political contexts that mediate, limit and provide opportunity for access and engagement with digital health technologies and the data they generate. As Cohn (2014: 157) observes: ‘a great wave of research over the last two decades attempting to develop techniques and evidence of behavioural change has proved to have surprisingly limited success’. To this end, a new trajectory of research must explore how cultures, practices and relations of power, shape access, use and engagement with digital health technologies, or else risk replicating or exacerbating existing inequalities.

In the few years, since England's creation of NHS Digital, a great deal more discussion has taken place. For example, in 2017, NHS Digital launched a wide consultation with digital developers to strategically involve itself within the design of third‐party platforms, not as co‐owners or co‐developers, but as an organisation interested in elevating the efficacy and research underpinning of such applications. As well, pilots are underway to launch a single NHS mobile app for patients to use, through which they can check symptoms, book and manage appointments with GPS, order repeat prescriptions, view their medical record, register as an organ donor, and choose whether and how the NHS uses their data (NHS Digital 2018). While there is a great appeal of a single point of access to digital health care, it will be crucial to monitor behaviours around adoption of mobile health applications, else it risks making a dramatic and detrimental impact on the improvement of health inequalities.

## Notes

1 The governance of healthcare within the United Kingdom is devolved across England, Northern Ireland, Scotland and Wales. Responsibility for public health care in each of these countries lies with respective governments.

2 The project ‘the digital health generation: the impact of healthy lifestyle technologies on young people's learning, identities and health practices’ was funded by the Wellcome Trust – 2017–2019.

3 ‘NHS Citizen aimed to “ensure that people and communities have an increasing say in health policy development; and how NHS services are commissioned, designed and delivered”’ https://www.england.nhs.uk/participation/get-involved/how/nhs-citizen/
